# The developing relationship between circadian rhythm and heart rate variability in premature infants

**DOI:** 10.1007/s10286-026-01216-1

**Published:** 2026-05-27

**Authors:** R. B. Govindan, Nickie N. Andescavage, Sudeepta Basu, Jonathan Murnick, Julius Ngwa, Catherine Limperopoulos, Adre J. du Plessis

**Affiliations:** 1 Prenatal and Transitional Pediatrics, The Zickler Family Prenatal Pediatrics Institute, Children’s National Hospital, 111 Michigan Ave NW, Washington, DC, 20010 USA; 2 The Developing Brain Institute, Children’s National Hospital, Washington, DC, USA; 3 Department of Pediatrics, The George Washington University School of Medicine, Washington, DC, USA; 4 Division of Neonatology, Children’s National Hospital, Washington, DC, USA; 5 Division of Diagnostic Imaging and Radiology, Children’s National Hospital, Washington, DC, USA; 6 Neurology School of Medicine and Health Sciences, The George Washington University, Washington, DC USA

Dear sir,

Heart rate variability (HRV) is a widely used tool to study autonomic function [13]. In low-risk term newborns, HRV metrics increase during the first 6 h of life and plateau after ~12 h [7]. In preterm infants, HRV is influenced by both morbidities [1, 4, 10] and behavioral state, and is lower in quiet than in active states [11, 15]. What remains poorly understood is the maturation of HRV in the context of circadian rhythm, which is a combination of several ultradian cycles [8]. Maternal rhythms entrain fetal circadian rhythms [6]. Preterm birth, off-schedule milk feeding, and neonatal intensive care unit (NICU) care can disrupt sleep–wake cycles, leading to immature circadian rhythms [2].

In mature individuals, the circadian peak and nadir correspond to periods of wakefulness (active state) and sleep (quiet state), respectively. In preterm infants, it is important to understand whether the maturational trajectory of HRV is similar or different when measured at the peak (important for long-term development) and nadir of the circadian cycle. In our prior study [2], we demonstrated the feasibility of measuring circadian rhythm in preterm infants. On the basis of those findings, we identified periods corresponding to active and quiet states. We therefore hypothesize that HRV will differ when measured during active versus quiet states.

In this prospective observational study, we recruited preterm infants born at ≤ 36 weeks’ gestation between May 2020 and July 2023, who were transferred to our level IV NICU at < 14 days of life for advanced care for prematurity-related morbidities. Exclusion criteria included periventricular leukomalacia, intraventricular hemorrhage (grades III–IV) as identified by routine head ultrasound on admission, and confirmed or suspected genetic conditions. Per protocol, all infants underwent brain magnetic resonance imaging at term-equivalent age (Discovery MR750, GE Healthcare). A trained neuroradiologist (J.M.) used the Kidokoro system [5] to classify images as normal (0–3), mild (4–7), moderate (8–11), or severe (≥ 12). Relevant clinical variables were retrieved from the medical records. The study was approved by the Children’s National Institutional Review Board, and informed consent was obtained for all participants. Continuous heart rate (HR) data were retrieved in Health Level 7 (HL7) format at a sampling rate of 0.2 Hz from bedside monitors (IntelliVue MP70, Philips Healthcare, Andover, MA, USA) from admission to infants’ discharge.

For each infant, HR data were extracted from 6 a.m. to 6 a.m. the following day and divided into 20-min epochs. Within each epoch, HR below 50 beats per minute (bpm) or above 250 bpm was considered artifact and excluded. The remaining HRs in each 20-min epoch were averaged, and a cosine function with 24-h period was fitted to the resulting time series as follows [2, 12]: ${\mathrm{HR}}_{t}=A\mathrm{cos}\left(\frac{2\pi }{24}t+\phi \right)+M,$, with A being the amplitude, M the mean value of the ${\mathrm{HR}}_{t}$, and ϕ the location of the amplitude. The correlation coefficient and its corresponding *P*-value were calculated between the fitted rhythm and ${\mathrm{HR}}_{t}$. A fit was considered reliable if *P* < 0.05. Days with more than 2 h of missing data were excluded. Peak and nadir were identified on days with a significant circadian fit. In addition, 20 min of raw HR data on either side of the circadian rhythm peak (totaling 40 min) and 20 min of data centered on the circadian rhythm nadir were extracted for analysis. The HRV metrics were characterized as the standard deviation (STD) of 40-min HR around peak $\left({\mathrm{STD}}_{\mathrm{Peak}}\right)$ and nadir $({\mathrm{STD}}_{\mathrm{Nadir}})$, respectively. Owing to the low temporal resolution, respiratory sinus arrhythmia was not analyzed. At group level, to construct trajectories of S${\mathrm{TD}}_{\mathrm{Peak}}$ and ${\mathrm{STD}}_{\mathrm{Nadir}}$ values were averaged every postmenstrual age (PMA: age at birth plus age since birth) week. For postnatal age (PNA: time since birth), the trajectories of S${\mathrm{TD}}_{\mathrm{Peak}}$ and ${\mathrm{STD}}_{\mathrm{Nadir}}$ values were averaged every PNA week in three different birth gestational age (GA) categories: (1) GA ≤ 27 (second half of the second trimester); (2) 28 ≥ GA ≤ 33 (early third trimester); and (3) GA > 33 weeks (late third trimester).

Normally distributed variables are reported as mean (STD), and non-normally distributed variables as median (minimum, maximum). The ordinal data were represented as counts (percentages). The changes in S${\mathrm{TD}}_{\mathrm{Peak}}$ and ${\mathrm{STD}}_{\mathrm{Nadir}}$ with both PMA and PNA were studied using independent linear mixed-effects models with random intercept and random slope terms clustered by subjects. Models included ventilator use, medications, pressors, caffeine, and antibiotics as time-varying covariates, with brain injury scores and sex as invariant covariates. For the PNA model, GA at birth was included as a static covariate. The final models were selected on the basis of the lowest Akaike information criterion (AIC) values. To characterize the difference in the HRV maturation between peak and nadir periods, we also developed matched logistic regression models. In these models, S${\mathrm{TD}}_{\mathrm{Peak}}$ and ${\mathrm{STD}}_{\mathrm{Nadir}}$ calculated for each day were paired and studied as a function of PMA and PNA using independent models. These models included  × $({\mathrm{Peak}}-{\mathrm{Nadir}})$  $Age \star ({\mathrm{Peak/Nadir}})$ interaction terms and only the dynamic covariates used in the linear mixed-effects models. *P* < 0.05 was considered statistically significant.

We enrolled 66 premature infants with a mean birth GA of 29.76 (3.61) weeks, with equal numbers of males and females. The median PNA at recruitment was 0 (0, 7) days, 16 (24%) required ventilator support, 44 (66%) received caffeine treatment for prematurity-related apnea and bradycardia, and 18 (27%) developed an infection and received antibiotics. The median 1-min Apgar score was 5 (4, 8), and 18 infants (27.27%) had a clinically significant patent ductus arteriosus. The Kidokoro scoring system revealed that 33 infants (61%) had no brain injury, 19 (35%) had mild injury, 2 (3.7%) had moderate injury, and none had severe injury. On admission ultrasound, 41 infants (74.55%) had no interventricular hemorrhage (IVH), 3 (5.45%) had unilateral grade I, 5 (9.1%) unilateral grade II, 1 (1.82%) bilateral grade I, and 6 (10.91%) bilateral grade II IVH.

HRV data were available for 542 days. In both the PMA and PNA models, the AIC was lower for models with random intercept and random slope terms. PMA was significantly positively associated with ${\mathrm{STD}}_{\mathrm{Peak}}$ (estimate: 0.52 bpm/week, *P* = 5.55 × 10^–15^) and ${\mathrm{STD}}_{\mathrm{Nadir}}$ (estimate: 0.39 bpm/week; *P* = 1.44 × 10^–8^), with a higher magnitude for the peak period than the nadir period (Fig. 1a). In addition, PNA was significantly associated with ${\mathrm{STD}}_{\mathrm{Peak}}$ (estimate: 0.55 bpm/week; *P* = 9.52 × 10^–16^) and ${\mathrm{STD}}_{\mathrm{Nadir}}$ (estimate: 0.39 bpm/week; *P* = 2.78 × 10^–7^), which is similar to the results obtained for PMA-based variation. Furthermore, in the PNA model, GA was associated with ${\mathrm{STD}}_{\mathrm{Peak}}$ (estimate: 0.44 bpm/week; *P* = 2.00 × 10^–5^) and ${\mathrm{STD}}_{\mathrm{Nadir}}$ (estimate: 0.33 bpm/week; *P* = 5.80 × 10^–4^), and none of the other covariates were significant in both PMA and PNA models. The results of the matched logistic regression analysis showed that PMA ×(Peak/Nadir) (estimate: 0.01; *P* = 0.01) and PNA × (Peak/Nadir) - interactions (estimate: 1.24 × 10^–2^; *P* = 6.87 × 10^–3^) were significant, indicating that the HRV maturation is different for peak and nadir periods.

**Fig. 1:**
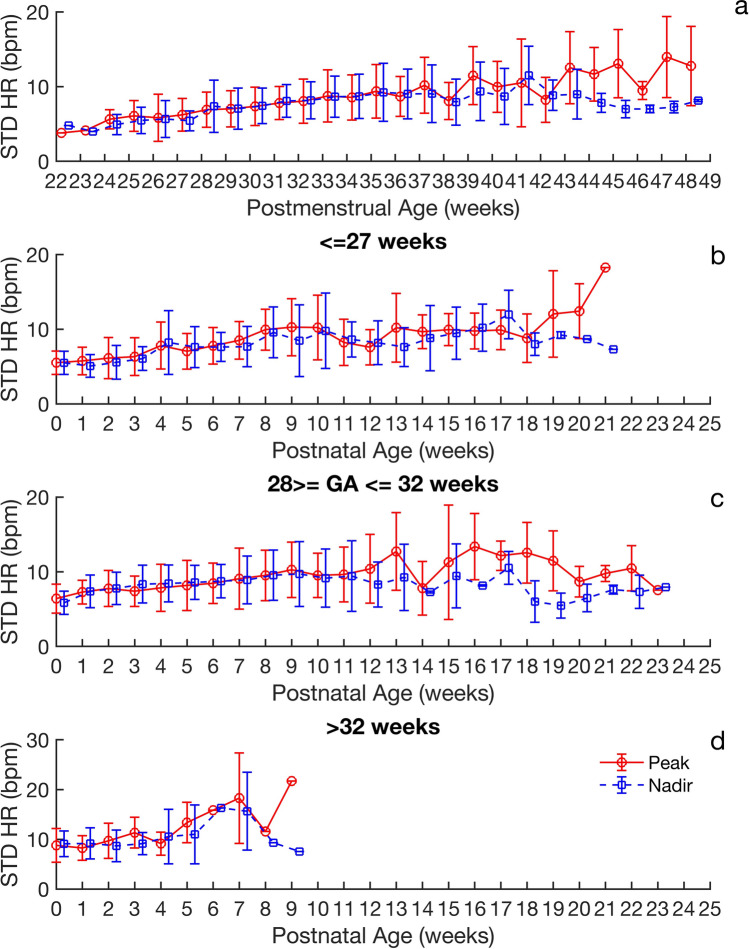
Trajectories of the standard deviation of heart rate (STD HR in beats per minute [bpm]) measured at the peak and nadir of the circadian rhythm are shown as a function of: **a** postmenstrual age, and **b**–**d** postnatal age across three gestational age-at-birth categories: **b** ≤ 27 weeks, **c** 28–32 weeks, and **d** > 32 weeks

HRV increased with PMA at both circadian peak and nadir (Fig. 1a), plateauing at ~35 weeks during the nadir. With PNA, HRV similarly increased but plateaued by ~8 weeks during the nadir, most notably in the 28–33-week group (Fig. 1b–d).

Infants experience approximately six to eight ultradian cycles of 50–90 min each over the course of each day [8]. Each cycle is composed of active and quiet sleep states. Consistent with previous studies in premature infants, we observed higher HRV during the active state compared with the quiet state [15]. We speculate that the observed plateau in HRV during the circadian nadir beginning around 8 weeks of age may represent an increase in ultradian cycle duration and a clearer segregation of sleep cycles within the 40-min analysis window used in this study. This observation is supported by electroencephalogram-based assessments of sleep–wake cycling [9]. Further studies are needed to confirm and better characterize sleep states in this cohort. The emergence of distinct states around 35 weeks PMA aligns with evidence that organized sleep–wake patterns develop by ~36 weeks PMA, with earlier patterns considered irregular “coincidental states”[14]. Our findings imply the link between autonomic development and circadian emergence.

This study has several limitations. We utilized low-resolution HR data, which limited our ability to apply advanced HRV techniques. Nonetheless, the STD of HR used in this study provided a reliable measure to capture maturation-related changes in variability. On the basis of prior literature, circadian peak and nadir were assumed to represent active and quiet states [11]. Future studies will characterize ultradian rhythms using HR [3, 11] and perform state-dependent variability analysis. Covariates such as feeding and infant handling may contribute to the variance in the STD of HR. We will consider these factors more rigorously in future studies.

Our analysis shows that autonomic maturation in preterm infants differs between circadian peak and nadir, plateauing in the quiet state after ~8 weeks, possibly due to lengthening of the ultradian cycle and increased predominance of quiet sleep at the nadir.

## Data Availability

The data will be shared on reasonable request to the corresponding author.
